# Comprehensive analysis of enzymatic browning in hazelnuts (*Corylus avellana* L.): influence of physical-biochemical, cultivar and agroclimatic parameters on nut development

**DOI:** 10.3389/fpls.2026.1816194

**Published:** 2026-05-28

**Authors:** Gabriela Gavilán-CuiCui, Daniela Padilla-Contreras, Antonieta Ruiz, Felipe González, Filis Morina, Cristian Meriño-Gergichevich

**Affiliations:** 1 Doctoral Program in Natural Resources Sciences, Universidad de La Frontera, Temuco, Chile; 2 Laboratory of Physiology and Plant Nutrition for Fruit Trees, Faculty of Agricultural Sciences and Environment, Universidad de La Frontera, Temuco, Chile; 3 Laboratory of Soil Fertility, Faculty of Agricultural Sciences and Environment, Universidad de La Frontera, Temuco, Chile; 4 Scientific and Technological Bioresources Nucleus (BIOREN-UFRO), Universidad de La Frontera, Temuco, Chile; 5 Department of Chemical Science and Natural Resources, Universidad de La Frontera, Temuco, Chile; 6 Czech Academy of Sciences, Biology Centre, Institute of Plant Molecular Biology, Laboratory of Plant Biophysics & Biochemistry, Ceske Budejovice, Czechia; 7 Master Program in Fruitculture, Faculty of Agricultural Sciences and Environment, Universidad de La Frontera, Temuco, Chile; 8 Department of Agricultural Production, Faculty of Agricultural Sciences and Environment, Universidad de La Frontera, Temuco, Chile

**Keywords:** browning, peroxidase, polyphenol oxidase, polyphenols, total phenolic content

## Abstract

Enzymatic browning (EB) is a physiological disorder that reduces the quality and commercial value of hazelnuts (*Corylus avellana* L.), associated with oxidative processes mediated by polyphenol oxidase (PPO) and peroxidase (POD), whose expression depends on the content of phenolic compounds, the phenological state of the nut, and agroclimatic conditions. The objective of this study was to analyze EB using physical-biochemical parameters, varietal response, and environmental influence, evaluating the incidence of EB, total phenolic content (TPC), and PPO and POD activity in the shell and kernel. The trial was conducted in two consecutive seasons, S1 (2022/2023) and S2 (2023/2024), in two contrasting locations in La Araucanía (Nueva Imperial and Radal), considering four phenological stages of the nut (BBCH 721–89). Industrial parameters showed higher quality in Barcelona, with larger nuts, better yield, and more protective shells. In S2, environmental restrictions limited nut filling and increased the risk of EB, especially in Tonda di Giffoni (TDG). The incidence of EB was low in S1 and increased in S2, mainly in BBCH 85, with increases of up to 392% in TDG in shell and kernel, higher in Radal and towards maturity. Likewise, TPC, PPO, and POD showed more intense responses in S2, with increases of 54–115%, 188%, and 410–529%, respectively, evidencing greater oxidative susceptibility of TDG in Radal. Overall, the results confirm that EB depends on complex multifactorial interactions, providing a basis for optimizing production management and varietal selection under increasing climate variability.

## 1 Introduction

Hazelnut (*Corylus avellana* L.) is one of the most important nuts crops worldwide, widely valued for its nutritional and functional properties and its industrial versatility (Poșta et al., 2022). Production is mainly concentrated in the northern hemisphere, with Turkey, Italy, the United States, Georgia, and Azerbaijan as the main producers, accounting for more than 90% of the global hazelnut yield (International Nuts and Dried Fruits Council, 2025). In recent decades, countries in the southern hemisphere, especially Chile, have increased their production and export of hazelnuts, taking advantage of their seasonality and favorable agroclimatic conditions (Oficina de Estudios y Políticas Agrarias (ODEPA), 2025). The expansion of this crop has positioned hazelnuts as a strategic alternative for national nut production, especially in the south-central region of the country (Oficina de Estudios y Políticas Agrarias (ODEPA), 2025). However, one of the main challenges facing the sector is enzymatic browning (EB), a defect that compromises the visual, sensory, and commercial quality of the nut, limiting its acceptance and value in the market (Gavilán-CuiCui et al., 2025).

The EB in hazelnut is an oxidative reaction catalyzed by various isoforms of polyphenol oxidases (PPO; EC 1.14.18.1) and peroxidases (POD; EC 1.11.1.7), which oxidize phenolic compounds and generate brown pigments (Zhou et al., 2015; Gheysarbigi et al., 2020). This phenomenon affects the shell and kernel, deteriorating their appearance, texture, and overall quality, with a negative impact on their commercial value (Gavilán-CuiCui et al., 2025). EB initiates when kernels attain 50-70% of final size, manifesting as brownish exudates and shell softening, which progress inward, causing abnormal moisture and discoloration (Olsen et al., 2013). Oxidation is activated by exposure to oxygen (O_2_), due to mechanical damage, ripening, or senescence, promoting the action of PPO and POD on catechins, hydroxycinnamic acids, and flavonoids, forming o-quinones that polymerize into melanins responsible for browning (Gavilán-CuiCui et al., 2025). Its severity depends on the time of occurrences: at the end of the summer, it is partial; in spring, it can compromise the entire nut season and make post-harvest handling difficult (Olsen et al., 2013).

Recent studies have shown that the incidence of EB depends on the content and type of phenolic compounds, as well as by enzymatic activity, which vary significantly depending on the cultivar, tissue structure, and phenological stage of the nut (Persic et al., 2017; Serra et al., 2021; Gavilán-CuiCui et al., 2025). In this context, differential distribution of phenolic compounds plays a key role as substrates in EB reactions, and their differential distribution among nut structures may largely explain the variability in susceptibility to this phenomenon (Fan et al., 2021; Serra et al., 2021). For example, it has been observed that hazelnut shells contain two to three times more total phenolic content (TPC) than the kernel, with values ranging from 159–730 mg gallic acid equivalents (GAE) 100 g^−1^ dry weight (DW) and 70–478 mg GAE 100 g^−1^ DW, respectively. The large variation in TPC content is not only due to the structure of the nut, but also due to genetic factors and environmental conditions (Di Michele et al., 2021; Meriño-Gergichevich et al., 2021; Gavilán-CuiCui et al., 2024). In turn, during the final stages of nut development, a sustained increase in PPO and POD activity has been reported, which coincides with a higher frequency and intensity of visible browning (Pan et al., 2021; Wang et al., 2025). This physiological response can be intensified by external factors, as agroclimatic variables such as temperature, solar radiation, relative humidity, and water stress directly influence the biosynthesis of phenolic compounds and the expression of genes related to antioxidant pathways and oxidative defense mechanisms (Shi et al., 2017; Guizani et al., 2019; Gavilán-CuiCui et al., 2024). Thus, EB is the result of the dynamic interaction between structural, biochemical, and environmental factors, whose manifestation varies depending on the cultivar (Yuan et al., 2019). In Chile, Barcelona and Tonda di Giffoni (TDG) differ in shell porosity and thickness, which impacts O_2_ penetration. These differences, together with variations in phenolic compound content and oxidative enzyme activity, jointly determine their variable susceptibility to EB (Gavilán-CuiCui et al., 2024, Gavilán-CuiCui et al., 2025).

In this context, the following hypothesis is proposed: enzymatic browning in hazelnuts is conditioned by interactions between physical-biochemical parameters, varietal responses, and agroclimatic factors, generating significant differences in the incidence and severity of EB in the shell and kernel structures throughout nut development, which vary according to the production season and contrasting locations. Given the above, the present study aims to comprehensively analyze EB in two varieties of hazelnuts, evaluating physical-biochemical parameters, varietal responses, and agroclimatic conditions. To this end, physical parameters associated with browning will be evaluated, such as the quality components and incidence of EB. In addition, total phenolic content will be quantified and the enzymatic activities of PPO and POD in the shell and kernel will be determined during different phenological stages of the nut, in two production seasons and two contrasting locations. This approach will provide a deep understanding of the processes that affect EB in Chilean hazelnuts, generating key information to optimize management, nut quality, and varietal selection, thereby contributing to strengthening the competitiveness of the sector.

## 2 Materials and methods

### 2.1 Experimental sites and collection of samples

**Figure 1:**
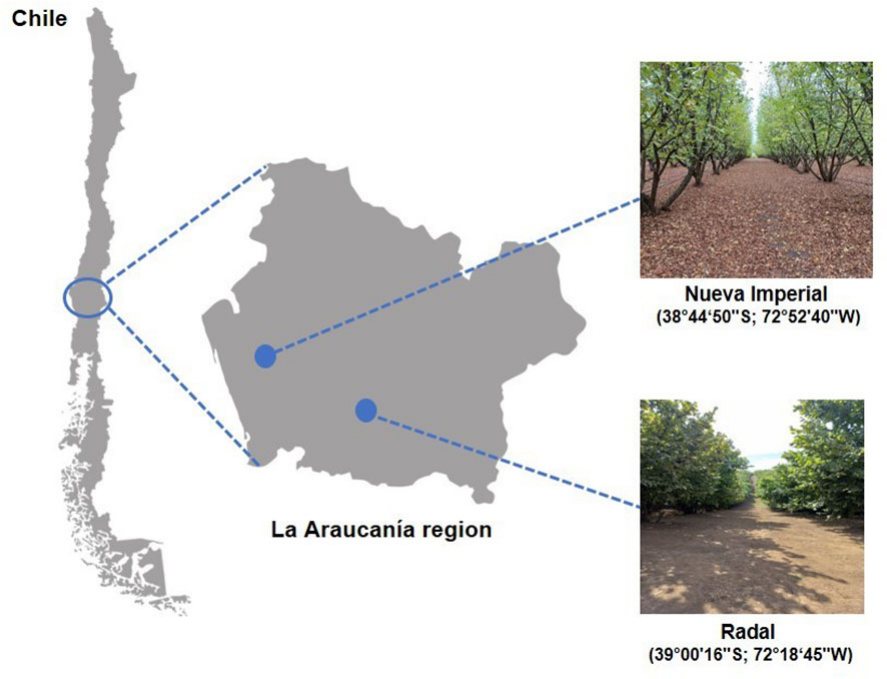
Experimental site, commercial orchards of Nueva Imperial (38°44′42″ S; 72°52′45″ W) and Radal (39°01′06″ S, 72°19′11″W), La Araucanía Region, Chile.

This study was conducted during two production seasons, 2022/2023 (S1) and 2023/2024 (S2). Hazelnuts were collected from the Barcelona and TDG cultivars from commercial orchards located in Nueva Imperial (38°44′42″ S; 72°52′45″ W; altitude: 20 m above sea level) and Radal (39°01′06″ S, 72°19′11″ W; altitude: 168 m above sea level), respectively, in La Araucanía Region, Chile (**Figure 1**).

Both commercial orchards were managed under standard agronomic conditions for the region, including supplemental irrigation during the growing season via a drip irrigation system with emitters supplying water at a rate of 2.0 L h^−1^. Irrigation scheduling was adjusted according to crop water requirements and local climatic conditions.

The climatic conditions of both locations during the seasons evaluated (S1 and S2) including precipitation (mm), temperature (°C), relative humidity (%), and solar radiation (MJ m^–2^), were obtained from Agromet INIA weather stations installed at each experimental site (https://agrometeorologia.cl/).

**Figure 2:**
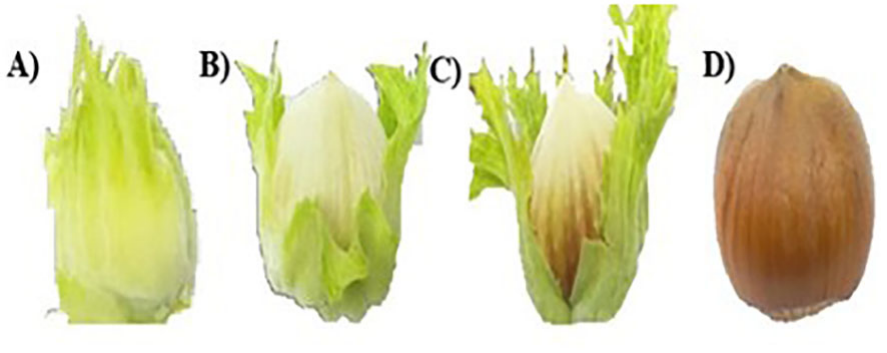
Representation of the phenological development of the hazelnut (*Corylus avellana* L.) in four stages according to the Biologische Bundesanstalt, Bundessortenamt und Chemische Industrie (BBCH) scale: **(A)** 721; **(B)** 75; **(C)** 85; and **(D)** 89. The images document the progress of development during the 2022/2023 (S1) and 2023/2024 (S2) seasons at Radal and Nueva Imperial, La Araucanía Region, Chile.

In both locations, hazelnuts were harvested manually at four identified phenological stages, from the beginning of nut growth (721) to physiological maturity (89). The phases were defined according to the codes of the BBCH (Biologische Bundesanstalt, Bundessortenamt und Chemische Industrie) phenological scale, as described by Taghavi et al. (2022) for this species:

➢ BBCH stage 721 (December): corresponds to the beginning of kernel filling, reaching more than 15% of the final nut diameter (**Figure 2A**).
➢ BBCH stage 75 (January): characterized by immature, green nuts in rapid development. The stigmas have fallen and approximately 50% of the nuts have reached their final size (**Figure 2B**).
➢ BBCH stage 85 (February): more than 50% of the nuts show signs of maturity, with progressive hardening of the shell and brown coloration (**Figure 2C**).
➢ BBCH stage 89 (March): represents full nut maturity, with more than 95% of the nuts fully developed and ready for harvest, falling naturally to the ground (**Figure 2D**).

After manual harvesting, samples were carefully collected to avoid mechanical damage. Immediately afterwards, they were placed in thermal containers at 4–8 °C and transported within six hours to preserve their stability at Laboratory of Physiology and Plant Nutrition for Fruit Trees, Universidad de La Frontera. Subsequently, the corresponding analyses were performed as detailed in the respective sections.

### 2.2 Morphological parameters associated with industrial quality

Thirty-six experimental units were evaluated, corresponding to individual plants considered as independent units. Ten hazelnuts were selected from each plant as subsamples, on which various morphological characteristics of the whole nut, kernel, and shell were measured, following the protocol described by Gavilán-CuiCui et al. (2024). The variables evaluated were length (mm), width (mm), thickness (mm), weight (g), nut roundness index (NRI), kernel roundness index (KRI), and kernel yield (%). A digital caliper (CALDI-6MP, Truper SA de CVCDMX, Mexico City, Mexico) was used to measure length, thickness, width, and internal cavity, while weight was determined using an analytical balance (Model BA2204B, Biobase Meihua Trading Co. Ltd., Jinan, Shandong, China). The nut roundness index (NRI), kernel roundness index (KRI), and kernel yield (%) were calculated as described by Gavilán-CuiCui et al. (2024).

### 2.3 Determinations of enzymatic browning incidence

The incidence of enzymatic browning (EB) was assessed in each hazelnut cultivar, considering four phenological stages and two production seasons. To do this, the methodology described by Pan et al. (2021) was adapted, incorporating some modifications. Ninety hazelnuts per cultivar were randomly selected, cracked open, and visually inspected for signs of EB. The browning index was determined by observing visible symptoms on the shell and kernel. Subsequently, the number of hazelnuts with browning was recorded and the index was calculated using the following formula:


$$\text{Enzymatic browning Index}\left(\%\right)=\frac{\text{Number of browning nuts}}{\text{Total number of nuts}}\times 100$$

### 2.4 Extraction of total phenolic compounds

To obtain phenolics extracts corresponding to the different phenological stages, a standardized extraction protocol was applied, optimized to ensure reproducibility and efficiency in the recovery of bioactive compounds. Shell samples were processed at all phenological stages analyzed, while kernel tissue extraction was performed from stage 75 onwards, due to the limited availability of biological material in the early stages of development. In each case, 0.5 g of shell and kernel powder were homogenized with 3 mL of the corresponding solvent under controlled conditions of amplitude and extraction time, as detailed in **Table 1**.

**Table 1:** Standardized extraction parameters for shell and kernel samples at four phenological stages of nuts development.

Phenological stages*	Nut material	Methanol: water (mL)	Amplitude (%)
721	Shell	90:10	60
	Kernel	--	--
75	Shell	75:25	80
	Kernel	50:50	80
85	Shell	90:10	80
	Kernel	50:50	80
89	Shell	75:25	80
	Kernel	50:50	60

*Biologische Bundesanstalt, Bundessortenamt und Chemische Industrie (BBCH) (Taghavi et al., 2022). Amplitude (%) represents the percentage of ultrasonic power applied during the ultrasound-assisted extraction process, according to the operating conditions of the equipment.

Subsequently, the mixtures were subjected to constant stirring for 10 min to promote the solubilization of the compounds of interest. After this stage, the samples were centrifuged at 10,000 × g for 10 min at 4 °C. The supernatant obtained was carefully recovered, protected from light, and stored at −80 °C until further analysis. The crude extract obtained was used to determine the phenolic compounds, as described in the following section.

#### 2.4.1 Total phenolic content

The TPC quantification was performed using the Folin-Ciocalteu method, with adaptations according to Gavilán-CuiCui et al. (2024). To this end, the following were added to each reaction microtube in the following order: 15 µL of sample or standard, 750 µL of deionized water, 75 µL of Folin-Ciocalteu reagent, 300 µL of 7% w/v sodium carbonate, and 360 µL of additional deionized water. The mixtures were incubated in the dark at 20 °C for 30 min. Subsequently, 250 µL was transferred to a 96-well plate, and the absorbance was measured at 750 nm using a SYNERGY HTX microplate reader (BioTek Instruments, Winooski, VT, USA). The calibration curve was generated with gallic acid (GA) at concentrations of 100, 200, 300, 400, and 500 mg L^−1^, and the results were expressed as gallic acid equivalents (GAE).

### 2.5 Extraction for enzymatic determinations

To obtain enzyme extracts from shell and kernel samples, 0.3 g of powdered tissue was weighed and immediately frozen in liquid nitrogen to preserve enzyme integrity. The samples were then homogenized in 0.9 mL of 0.1 M potassium phosphate buffer (pH 7.0). The mixture was centrifuged at 13,000 × g for 15 min at 4 °C (Lab. Companion centrifuge, Korea), and the supernatant obtained was stored at -80 °C until use. In order to eliminate interference caused by phenolic compounds, the extracts were pretreated with polyvinylpyrrolidone (PVPP). To do this, 10 mg of PVPP was added to 400 μL of enzyme extract, stirred manually, and centrifuged at 12,000 × g for 10 min at 4 °C. The clarified supernatant was used for both enzyme determinations and total protein quantification using the Bradford method (Bradford, 1976). All spectrophotometric readings were performed on a microplate reader (Synergy HTX, UV-visible, Biotek, Winooski, VT, USA). Enzyme activity was expressed as units per milligram of protein (U mg^−1^ protein), considering one enzyme unit (U) as the change in absorbance of 0.001 units per min under the assay conditions.

### 2.6 Determination of enzymatic activities

#### 2.6.1 Polyphenol oxidase

Enzyme activity was quantified using a protocol based on Sun et al. (2018), with modifications. The reaction mixture (220 µL) consisted of 40 µL of phosphate buffer (0.1 M, pH 7.0) and 160 µL of catechol (1 mM). The mixture, without enzyme extract, was preincubated at 37 °C for 3 min. The reaction was initiated by adding 20 µL of crude enzyme extract. The increase in absorbance was recorded at 420 nm for 5 min, with readings taken every 60 s. Enzyme activity was expressed in PPO units (U), where 1 U corresponds to a change of 0.001 in absorbance per minute (ΔAbs_420_/min), associated with the formation of the oxidized product of catechol.

#### 2.6.2 Peroxidase

Enzyme activity was quantified using a protocol based on Sun et al. (2018), with modifications. The reaction mixture (117 µL) consisted of 35 µL of phosphate buffer (0.1 M, pH 7.0), 25 µL of p-phenylenediamine (1%, w/v), 12 µL of hydrogen peroxide (1.5%, v/v), and 25 µL of tropolone (1%, w/v). The mixture, without the enzyme extract, was preincubated at 28 °C for 3 min. The reaction was initiated by adding 20 µL of crude enzyme extract. The increase in absorbance was measured at 485 nm for 5 min, with readings taken every 60 s. Enzyme activity was expressed in POD units (U), where 1 U corresponds to a change of 0.001 in absorbance per minute (ΔAbs_485_/min), associated with the formation of the oxidized product of p-phenylenediamine.

### 2.7 Experimental design and statistical analysis

All statistical analyses were performed using R software (v.4.2.1). Normality and homogeneity of variances were verified beforehand. For each cultivar, the data were analyzed separately by type of nut material (shell and kernel) using three-way analysis of variance (ANOVA), considering the phenological stage (PS), season (S), and location (L) as factors to evaluate their effects on physical, antioxidant, and enzymatic parameters. When significant differences were detected (p<0.05), the means were compared using the Tukey HSD test with the “agricolae” library (v.1.3-5). The results are presented as means ± standard error, and statistically significant differences are indicated by different lowercase letters. Multivariate relationships between the evaluated parameters were explored using principal component analysis (PCA).

## 3 Results

### 3.1 Weather conditions

**Figure 3:**
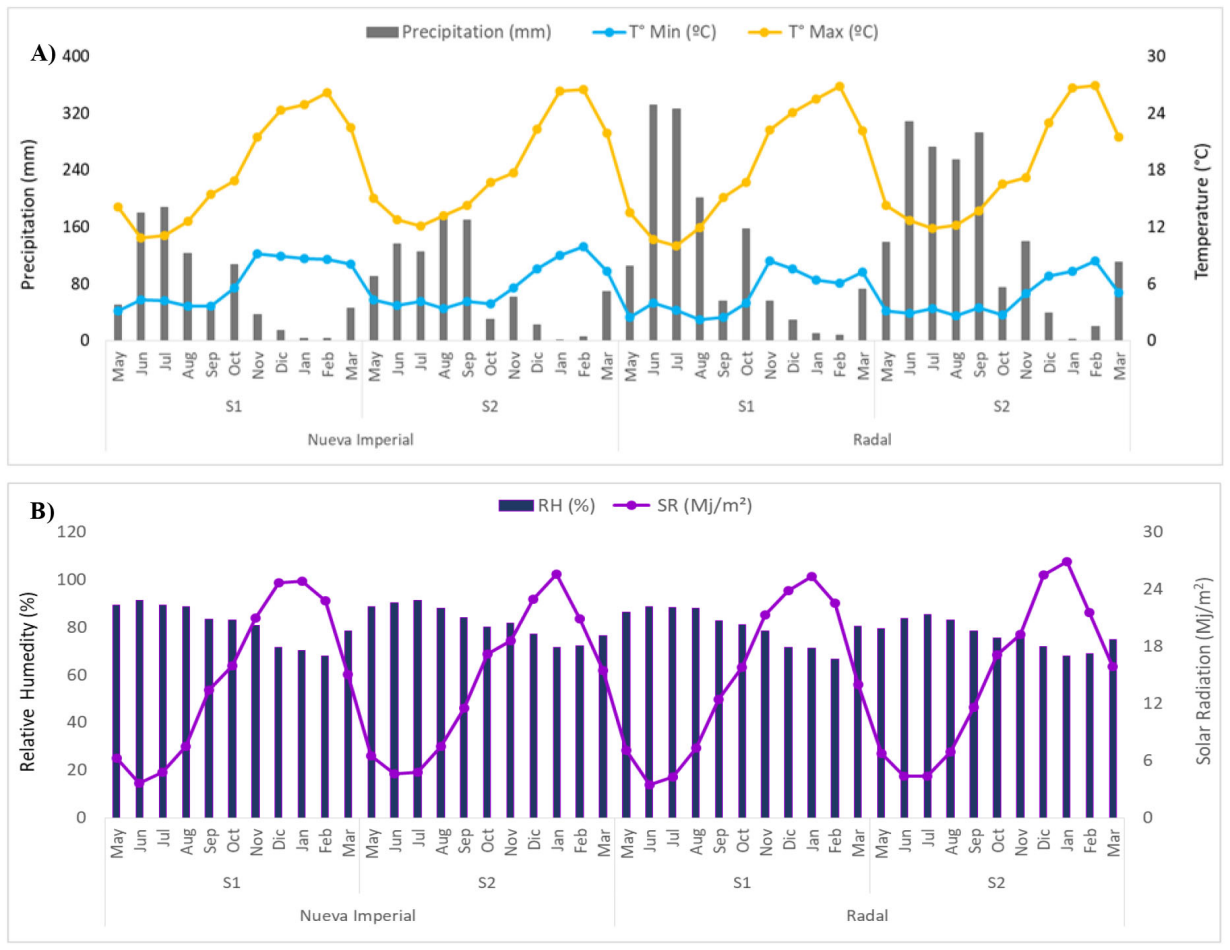
Weather conditions in Nueva Imperial and Radal during the 2022/2023 (S1) and 2023/2024 (S2) growing seasons: **(A)** precipitation (mm) and temperature (°C); **(B)** relative humidity (%) and solar radiation (MJ m^−^²). Data obtained from Agromet INIA (https://agrometeorologia.cl/).

In S1, Nueva Imperial, the highest precipitation was recorded during the winter, reaching its maximum in July (187.8 mm), while values decreased sharply towards summer, with a minimum in February (2.8 mm). In Radal, during S1, precipitation was higher and more concentrated, with maximums in June (331.2 mm) and July (326.3 mm), and a broader distribution that included significant amounts in autumn (104.8 mm in May) and spring (157.3 mm in October), while the lowest precipitation values were recorded in February (7.6 mm). Maximum temperatures during S1 reached their highest value in February, with 26.2 °C in Nueva Imperial and 26.8 °C in Radal, while minimum temperatures were lower in Radal (2.2 °C in August) than in Nueva Imperial (3.1 °C in May) (**Figure 3A**). Solar radiation showed a defined seasonal pattern during S1, with maximums in summer, reaching 24.9 MJ m^−^² in January in Nueva Imperial and 25.4 MJ m^−^² in Radal, and minimums in June (3.6 and 3.5 MJ m^−^², respectively). Relative humidity remained high during winter in S1, with maximum values in June (91.5% in Nueva Imperial and 88.7% in Radal), and decreased towards summer, reaching minimums in February (68.2 and 66.7%, respectively) (**Figure 3B**).

During S2, Nueva Imperial had its highest precipitation in August (175.9 mm), with high values also in September (169.6 mm), while the lowest precipitation was recorded in summer, particularly in January (0.6 mm) and February (5.3 mm). In Radal, during S2, precipitation was more intense and concentrated in winter and early spring, reaching a maximum in June (308 mm), followed by a marked decrease in October (74.6 mm) and minimum values in January (2.3 mm) and February (19.5 mm). Maximum summer temperatures exceeded 26 °C in both locations during S2, with values of 26.5 °C in Nueva Imperial and 26.9 °C in Radal in February, while minimum temperatures were recorded in winter, being slightly lower in Radal (2.6 °C in August) than in Nueva Imperial (3.4 °C) (**Figure 3A**). Solar radiation during S2 peaked in January, with 25.6 MJ m^−^² in Nueva Imperial and 26.9 MJ m^−^² in Radal and decreased to minimum values in June (4.6 and 4.4 MJ m^−^², respectively). The relative humidity of S2 showed a seasonal pattern similar to that of S1, with winter maximums (91.4% in Nueva Imperial and 85.6% in Radal in July) and summer minimums in February (72.2 and 69%, respectively) (**Figure 3B**).

### 3.2 Industrial parameters

The industrial quality parameters of the nut and shell of the Barcelona and TDG cultivars were significantly influenced by L, S, and PS, as well as by their interactions. Nut weight and dimensions were the variables most sensitive to these factors, while the roundness index (NRI) showed a more stable response to the conditions evaluated.

Barcelona nuts from S1 Nueva Imperial exhibited superior mass (3.55–3.81 g), length (18.35–21 mm), thickness (15.99–18.81 mm), and width (18.41–23.10 mm) between phenological stages BBCH 721 to 89, accompanied by an NRI close to 1.0, reflecting more symmetrical and balanced nuts. The shell had higher values for weight (2.02–2.68 g) and thickness (1.56–1.78 mm), indicating a more compact and protective structure. In Radal during S1, the weight increased progressively from 2.06 g (BBCH 721) to 3.51 g (BBCH 89), while the NRI remained between 0.96-1.05, with no significant differences. The shell thickness ranged from 1.57–1.90 mm, indicating nuts with a good ratio between axis and diameter. Barcelona in S2, Nueva Imperial, showed the lowest values for weight and dimensions in the early stages of development (BBCH 721), with an average weight of 0.69 g and a length of 10.78 mm, indicating limited initial development. However, towards the advanced stages (BBCH 85–89), the nuts significantly increased their weight to 3.5 g and shell thickness (1.91 mm), indicating more elongated nuts and greater shell lignification. In contrast, NRI showed no significant differences. In Radal during S2, the behavior was more restrictive, with nuts showing the lowest initial values for weight (0.41 g) and length (9.5 mm), as well as reduced shell thickness (0.77 mm). Towards maturity, weight and size recovered partially (2.5–3.5 g and 21 mm in length), with the latter reaching a value similar to that observed in Nueva Imperial (**Table 2**).TDG in S1, Nueva Imperial, showed higher values for nut weight (3.18-3.42 g), thickness (17.13-17.90 mm), and width (20.35-20.90 mm) in BBCH stages 75, 85, and 89 compared to BBCH 721. Likewise, the weight of the shell also showed significant differences between the stages of development, while the other variables of the nut, including length and NRI, as well as shell thickness, showed no statistical differences between the stages evaluated. In Radal during S1, it showed higher nut weight values (2.98-3.24 g) in BBCH 85 and 89 compared to BBCH 721 and 75. Likewise, the parameters of nut length (16.97-17.65 mm), thickness (16.68-16.96 mm), and width (19.02-19.51 mm) were higher from BBCH 75 to 89 compared to BBCH 721. The weight of shell statistical differences between stages of developments, while the NRI and shell thickness did not show significant differences. In S2, Nueva Imperial, TDG showed a marked reduction in weight and dimensions in the early stages (BBCH 721), with values of 0.45 g nut weight, 10.36 mm length, 6.84 mm thickness, and 12.33 mm width, along with a shell weight of 0.45 g, indicating limited initial development. However, towards maturity (BBCH 85–89), the nuts increased in weight to 3.45 g, length (19.0 mm), thickness (18.41 mm), and width (21.9 mm). Meanwhile, NRI and shell thickness showed no statistical differences. In Radal during S2, the lowest values of the parameters evaluated were recorded at BBCH 721, with nut weight of 0.38 g, length of 8.45 mm, thickness of 6.03 mm, and width of 9.56 mm, along with a thin shell (0.38 mm), indicative of a delay in initial development. Towards BBCH 85, the weight partially recovered (2.89 g), and from BBCH 75 to 89, the parameters of nut length, thickness, width, and shell weight increased. Meanwhile, NRI and shell thickness showed no statistical differences (**Table 3**).The industrial parameters of the kernels of the Barcelona and TDG cultivars were significantly influenced by L, S, and PS, as well as by some of their interactions. Among the variables evaluated, kernel weight and certain physical dimensions proved to be the most sensitive to these factors, while the kernel roundness index (KRI) showed a more stable response, remaining close to unit values in most of the conditions analyzed.

**Table 2:** Industrial quality of nut and shells with enzymatic browning in the Barcelona cultivar evaluated at different phenological stages according to the BBCH scale (721, 75, 85, and 89) during the 2022/2023 (S1) and 2023/2024 (S2) in Nueva Imperial and Radal, La Araucanía region.

				Nut					Shell	
Cultivar	Locality	Season	BBCH	Weight	Length	Thickness	Width	NRI	Weight	Thickness
				g	mm				g	mm
Barcelona	Nueva Imperial	S1	721	2.62 ± 0.08e	18.35 ± 1.68abc	15.99 ± 2.38ab	18.41 ± 1.41ab	0.93 ± 0.04a	2.62 ± 0.08abc	1.78 ± 0.03abcd
			75	3.55 ± 0.03ab	19.45 ± 0.30abc	18.22 ± 0.49ab	20.31 ± 0.38ab	0.99 ± 0.02a	2.30 ± 0.05bcde	1.56 ± 0.07d
			85	3.57 ± 0.06ab	19.00 ± 0.81abc	18.65 ± 1.76ab	22.40 ± 2.31ab	1.08 ± 0.06a	2.02 ± 0.09cdefg	1.66 ± 0.04abcd
			89	3.81 ± 0.05a	21.00 ± 1.08a	18.81 ± 0.47ab	23.10 ± 0.69a	0.99 ± 0.02a	2.68 ± 0.38ab	1.75 ± 0.05abcd
		S2	721	0.69 ± 0.03g	10.78 ± 0.53d	8.65 ± 0.20c	13.14 ± 0.30cd	1.01 ± 0.04a	0.69 ± 0.03h	1.18 ± 0.09e
			75	3.20 ± 0.06bcd	20.03 ± 0.93abc	16.94 ± 0.64ab	20.00 ± 1.12ab	0.92 ± 0.06a	3.07 ± 0.10a	1.57 ± 0.05cd
			85	3.57 ± 0.02ab	19.92 ± 0.44abc	19.78 ± 0.17a	22.48 ± 0.49ab	1.06 ± 0.02a	1.77 ± 0.05efg	1.85 ± 0.07abcd
			89	3.51 ± 0.23abc	19.04 ± 0.32abc	17.68 ± 0.39ab	20.84 ± 0.48ab	1.01 ± 0.15a	1.66 ± 0.09fg	1.91 ± 0.10a
	Radal	S1	721	2.06 ± 0.10f	15.90 ± 1.27c	14.87 ± 1.50b	17.61 ± 1.47bc	1.02 ± 0.01a	2.06 ± 0.05cdef	1.90 ± 0.06ab
			75	2.83 ± 0.07de	16.37 ± 0.15bc	16.37 ± 0.24ab	18.13 ± 0.52abc	1.05 ± 0.02a	1.42 ± 0.08g	1.57 ± 0.03cd
			85	3.30 ± 0.02abcd	20.43 ± 0.49ab	18.34 ± 0.33ab	20.91 ± 0.60ab	0.96 ± 0.03a	2.68 ± 0.07ab	1.64 ± 0.07abcd
			89	3.51 ± 0.28abc	20.21 ± 0.92ab	18.66 ± 0.65ab	21.51 ± 1.18ab	0.99 ± 0.01a	1.87 ± 0.06defg	1.59 ± 0.06cd
		S2	721	0.41 ± 0.02g	9.53 ± 0.52d	7.52 ± 0.61c	10.51 ± 0.66d	0.94 ± 0.04a	0.41 ± 0.03h	0.77 ± 0.04f
			75	2.99 ± 0.08cde	18.94 ± 0.78abc	17.85 ± 0.51ab	20.66 ± 0.42ab	1.01 ± 0.03a	2.45 ± 0.04bcd	1.87 ± 0.03abc
			85	3.53 ± 0.05abc	20.83 ± 0.44a	17.65 ± 0.46ab	21.04 ± 0.63ab	0.92 ± 0.03a	1.73 ± 0.08efg	1.60 ± 0.06bcd
			89	2.49 ± 0.09ef	21.33 ± 0.37a	19.01 ± 0.27ab	22.05 ± 0.43ab	0.96 ± 0.01a	1.96 ± 0.12defg	1.92 ± 0.05a
Significance	L			***	NS	NS	*	NS	***	NS
	S			***	**	**	**	NS	***	**
	PS			***	***	***	***	NS	***	***
	L x PS			*	**	**	NS	*	***	**
	S x L			NS	NS	NS	NS	NS	NS	NS
	S x PS			***	***	***	***	NS	***	***
	S x L x PS			**	NS	NS	NS	NS	***	***

L, location; S, season; PS, phenological stage. Values represent the mean of ten subsamples per experimental unit ± standard error. Different letters indicate statistically significant differences (p<0.05) for each cultivar within each location, season, and phenological stage. NS: not significant; * p<0.05; ** p<0.01; *** p<0.001.

**Table 3:** Industrial quality of nut and shells with enzymatic browning in the Tonda di Giffoni (TDG) cultivar evaluated at different phenological stages according to the BBCH scale (721, 75, 85, and 89) during the 2022/2023 (S1) and 2023/2024 (S2) in Nueva Imperial and Radal, La Araucanía region.

				Nut					Shell	
Cultivar	Locality	Season	BBCH	Weight	Length	Thickness	Width	NRI	Weight	Thickness
				g	mm				g	mm
TDG	Nueva Imperial	S1	721	2.21 ± 0.06f	16.99 ± 1.52a	13.01 ± 0.90c	17.46 ± 0.12d	0.89 ± 0.05b	2.21 ± 0.09b	1.54 ± 0.05a
			75	3.18 ± 0.03bc	18.90 ± 0.26a	17.90 ± 0.28ab	20.9 ± 0.63ab	1.02 ± 0.03ab	2.06 ± 0.09bc	1.49 ± 0.10a
			85	3.12 ± 0.03cd	18.75 ± 0.07a	17.86 ± 0.07ab	20.51 ± 0.09abc	1.02 ± 0.01ab	1.83 ± 0.04cd	1.31 ± 0.07a
			89	3.42 ± 0.08ab	19.44 ± 0.46a	17.13 ± 0.47ab	20.35 ± 0.31abc	0.96 ± 0.03ab	1.85 ± 0.07cd	1.55 ± 0.07a
		S2	721	0.45 ± 0.04h	10.36 ± 0.63bc	6.84 ± 0.56de	12.33 ± 0.55f	0.92 ± 0.02ab	0.45 ± 0.04g	1.26 ± 0.08a
			75	2.86 ± 0.06d	19.00 ± 0.75a	15.77 ± 0.80b	19.05 ± 0.94bcd	0.91 ± 0.06ab	2.72 ± 0.11a	1.46 ± 0.04a
			85	2.99 ± 0.05cd	18.90 ± 0.59a	18.41 ± 0.39a	20.68 ± 0.25ab	1.03 ± 0.03ab	1.55 ± 0.05def	1.63 ± 0.08a
			89	3.45 ± 0.01a	19.00 ± 0.48a	17.26 ± 0.36ab	21.94 ± 0.40a	1.03 ± 0.03ab	1.76 ± 0.08cd	1.77 ± 0.07a
	Radal	S1	721	1.17 ± 0.09g	12.00 ± 0.52b	8.58 ± 0.65d	14.48 ± 0.60e	0.92 ± 0.02ab	1.28 ± 0.10f	2.27 ± 0.07a
			75	2.57 ± 0.05e	17.65 ± 0.31a	16.96 ± 0.22ab	19.51 ± 0.48bcd	1.03 ± 0.02a	1.78 ± 0.06cd	1.62 ± 0.06a
			85	2.98 ± 0.06cd	17.21 ± 0.23a	16.44 ± 0.44ab	19.05 ± 0.41bcd	1.03 ± 0.02a	1.44 ± 0.08ef	1.32 ± 0.07a
			89	3.24 ± 0.04abc	16.97 ± 0.81a	16.68 ± 0.67ab	19.02 ± 0.79bcd	1.05 ± 0.01a	1.58 ± 0.03def	1.52 ± 0.04a
		S2	721	0.38 ± 0.02h	8.45 ± 0.43c	6.03 ± 0.31e	9.56 ± 0.28g	0.92 ± 0.02ab	0.38 ± 0.01g	1.04 ± 0.03a
			75	2.34 ± 0.09ef	18.86 ± 0.55a	17.27 ± 0.74ab	20.24 ± 0.45abc	0.99 ± 0.04ab	1.63 ± 0.05de	1.59 ± 0.07a
			85	2.89 ± 0.09d	18.96 ± 0.28a	16.79 ± 0.14ab	18.42 ± 0.23cd	0.92 ± 0.01ab	1.43 ± 0.08ef	1.53 ± 0.07a
			89	2.13 ± 0.09f	18.44 ± 0.21a	17.32 ± 0.27ab	20.24 ± 0.16abc	1.01 ± 0.01ab	1.35 ± 0.03ef	1.85 ± 0.06a
Significance	L			***	***	***	***	NS	***	NS
	S			***	*	***	***	NS	***	NS
	PS			***	***	***	***	***	***	NS
	L x PS			***	**	***	***	NS	***	NS
	S x L			NS	**	NS	NS	NS	NS	NS
	S x PS			***	***	***	***	*	***	NS
	S x L x PS			***	NS	***	*	NS	***	NS

L, location; S, season; PS, phenological stage. Values represent the mean of ten subsamples per experimental unit ± standard error. Different letters indicate statistically significant differences (p<0.05) for each cultivar within each location, season, and phenological stage. NS: not significant; * p<0.05; ** p<0.01; *** p<0.001.

In Barcelona, during S1 in Nueva Imperial, kernel weight increased from BBCH 75 to BBCH 89. However, despite observing an upward trend associated with the progress of ripening, no statistical differences were detected in kernel dimensions, including length, thickness, width, KRI, and yield. In Radal, during S1, Barcelona showed a progressive and stable growth pattern, with an increase in kernel weight from 1.01 to 1.71 g, while the remaining physical variables, such as length, thickness, width, KRI, and yield, showed no differences between phenological stages, suggesting balanced morphological development. During S2, in Nueva Imperial, Barcelona showed a significant decrease in the initial kernel values at BBCH 75, particularly in weight (0.91 g), thickness (1.97 mm), and width (3.19 mm), evidencing limited initial filling. However, as maturation progressed to BBCH 89, a partial recovery in kernel size and weight was observed (1.54 g, 14.72 mm thickness, and 16.44 mm width), suggesting an acceleration in final development possibly associated with restrictive environmental conditions during the early stages. In contrast, kernel length, KRI, and yield did not show significant differences between phenological stages. In Radal, during S2, kernel weight was consistently low, with an initial value of 0.59 g at BBCH 75, indicating a marked restriction in growth under drier conditions, although weight increased to 0.96 g at BBCH 89, the KRI remained close to 1.0, reflecting lighter kernels with lower density (**Table 4**).In TDG, during S1 in Nueva Imperial, kernel weight ranged from 1.06 to 1.59 g, showing an increase associated with the progress of ripening, while the other physical variables did not show significant differences between BBCH 75 and 89, despite exhibiting an upward trend. In Radal, during S1, kernel weight (0.77–1.43 g) and yield (30–44%) increased between BBCH 75 and 89. In contrast, length, thickness, and width did not show significant changes between the final stages, while the KRI remained close to 1.0, indicating a relatively stable and proportional kernel morphology. During S2, in Nueva Imperial, the TDG kernel showed a drastic reduction in BBCH 75 compared to BBCH 85 and 89, with minimum values for weight (0.51 g), length (3.80 mm), thickness (2.40 mm), width (2.66 mm), KRI (0.56), and yield (17.99%), reflecting severely limited initial filling. However, upon reaching BBCH 89, kernel weight increased to 1.39 g and yield to 40.29%, with a KRI of 0.94, although the nuts remained smaller in size compared to S1. In Radal, during S2, the evaluated parameters of the TDG kernel remained stable with respect to the initial condition, resulting in small kernels that were morphologically underdeveloped (**Table 5**).

**Table 4:** Industrial quality of kernels with enzymatic browning in the Barcelona cultivar evaluated at different phenological stages according to the BBCH scale (75, 85, and 89) during the 2022/2023 (S1) and 2023/2024 (S2) in Nueva Imperial and Radal, La Araucanía region.

				Kernel					
Cultivar	Locality	Season	BBCH	Weight	Length	Thickness	Width	KRI	Yield
				g	Mm				%
Barcelona	Nueva Imperial	S1	75	1.36 ± 0.07c	15.06 ± 1.34a	11.46 ± 0.27ab	12.59 ± 0.13ab	0.79 ± 0.07a	38.26 ± 5.22abc
			85	1.68 ± 0.02b	15.99 ± 1.04a	14.54 ± 1.42ab	15.90 ± 0.76a	0.95 ± 0.03a	47.06 ± 5.00ab
			89	1.99 ± 0.05a	16.01 ± 1.02a	15.53 ± 0.92a	15.61 ± 1.28a	0.97 ± 0.03a	52.35 ± 4.05a
		S2	75	0.91 ± 0.03e	4.10 ± 0.80b	1.97 ± 0.30c	3.19 ± 0.06b	0.63 ± 0.09a	28.39 ± 3.68bc
			85	1.46 ± 0.09bc	13.97 ± 2.18ab	13.86 ± 1.48ab	15.38 ± 1.48a	1.04 ± 0.02a	41.07 ± 4.92abc
			89	1.54 ± 0.07bc	14.03 ± 0.29ab	14.72 ± 0.31ab	16.44 ± 0.15a	1.08 ± 0.16a	43.90 ± 5.32ab
	Radal	S1	75	1.01 ± 0.06de	12.61 ± 0.56ab	11.47 ± 0.15ab	14.57 ± 0.38ab	1.03 ± 0.03a	35.61 ± 3.47abc
			85	1.50 ± 0.01bc	16.01 ± 0.62a	15.53 ± 1.59a	15.61 ± 1.24a	0.97 ± 0.08a	45.67 ± 1.63ab
			89	1.71 ± 0.04b	15.13 ± 0.39a	13.83 ± 0.99ab	16.62 ± 1.29a	1.00 ± 0.07a	48.85 ± 4.70ab
		S2	75	0.59 ± 0.01f	10.42 ± 3.47ab	6.82 ± 2.29bc	12.62 ± 4.49ab	0.93 ± 0.04a	19.96 ± 6.82c
			85	1.27 ± 0.07cd	12.70 ± 4.50ab	10.09 ± 3.65abc	13.93 ± 5.14ab	0.94 ± 0.33a	35.99 ± 2.05abc
			89	0.96 ± 0.02e	12.89 ± 3.25ab	10.08 ± 2.61abc	14.89 ± 3.76ab	0.96 ± 0.08a	38.62 ± 5.53abc
Significance	L			***	NS	NS	NS	NS	NS
	S			***	**	***	NS	NS	***
	PS			***	*	***	*	*	***
	L x PS			*	NS	NS	NS	NS	NS
	S x L			NS	NS	NS	NS	NS	NS
	S x PS			***	NS	NS	NS	NS	NS
	S x L x PS			NS	NS	NS	NS	NS	NS

L, location; S, season; PS, phenological stage. Values represent the mean of ten subsamples per experimental unit ± standard error. Different letters indicate statistically significant differences (p< 0.05) for each cultivar within each location, season, and phenological stage. NS: not significant; * p<0.05; ** p<0.01; *** p<0.001.

**Table 5:** Industrial quality of kernels with enzymatic browning in the Tonda di Giffoni (TDG) cultivar evaluated at different phenological stages according to the BBCH scale (75, 85, and 89) during the 2022/2023 (S1) and 2023/2024 (S2) in Nueva Imperial and Radal, La Araucanía region.

				Kernel					
Cultivar	Locality	Season	BBCH	Weight	Length	Thickness	Width	KRI	Yield
				g	mm				%
TDG	Nueva Imperial	S1	75	1.06 ± 0.08bcd	13.84 ± 0.41ab	11.06 ± 0.32ab	12.32 ± 0.34abc	0.84 ± 0.02a	33.55 ± 2.93abc
			85	1.38 ± 0.03ab	14.87 ± 0.56a	13.66 ± 0.02a	14.80 ± 0.55ab	0.95 ± 0.01a	44.16 ± 5.25ab
			89	1.59 ± 0.06a	13.89 ± 0.46ab	11.71 ± 0.45a	13.23 ± 0.47abc	0.89 ± 0.05a	46.47 ± 1.10a
		S2	75	0.51 ± 0.18e	3.80 ± 0.73c	2.40 ± 0.81c	2.66 ± 0.40d	0.56 ± 0.03b	17.99 ± 3.46d
			85	1.13 ± 0.19bcd	11.77 ± 3.23ab	10.69 ± 3.30ab	12.70 ± 3.63abc	0.99 ± 0.11a	37.85 ± 5.57abc
			89	1.39 ± 0.03ab	14.36 ± 1.36ab	12.11 ± 2.50a	15.00 ± 0.84ab	0.94 ± 0.02a	40.29 ± 3.02abc
	Radal	S1	75	0.77 ± 0.09de	11.77 ± 0.39ab	9.27 ± 0.43ab	10.61 ± 0.58bc	0.84 ± 0.02a	30.20 ± 3.95bcd
			85	1.18 ± 0.04bc	14.55 ± 0.23a	13.11 ± 0.43a	14.59 ± 0.37ab	0.95 ± 0.02a	39.59 ± 2.62abc
			89	1.43 ± 0.04ab	13.99 ± 0.36ab	13.37 ± 0.64a	15.96 ± 1.01a	1.04 ± 0.06a	44.32 ± 6.47ab
		S2	75	0.41 ± 0.07e	9.35 ± 2.42b	6.77 ± 1.77bc	8.05 ± 1.46c	0.79 ± 0.02ab	17.67 ± 6.25d
			85	1.00 ± 0.12cd	10.55 ± 2.79ab	9.28 ± 2.62ab	11.51 ± 3.54abc	0.98 ± 0.13a	34.87 ± 4.81abc
			89	0.56 ± 0.09e	12.04 ± 2.27ab	9.40 ± 1.83ab	13.62 ± 2.56ab	0.95 ± 0.05a	26.59 ± 5.13cd
Significance	L			***	NS	NS	NS	*	*
	S			***	***	***	***	NS	***
	PS			***	***	***	***	***	***
	L x PS			**	NS	NS	NS	NS	NS
	S x L			NS	NS	NS	NS	NS	NS
	S x PS			*	**	*	**	*	NS
	S x L x PS			***	**	**	**	*	NS

L, location; S, season; PS, phenological stage. Values represent the mean of ten subsamples per experimental unit ± standard error. Different letters indicate statistically significant differences (p< 0.05) for each cultivar within each location, season, and phenological stage. NS: not significant; * p<0.05; ** p<0.01; *** p<0.001.

### 3.3 Determinations of the incidence of enzymatic browning

**Figure 4:**
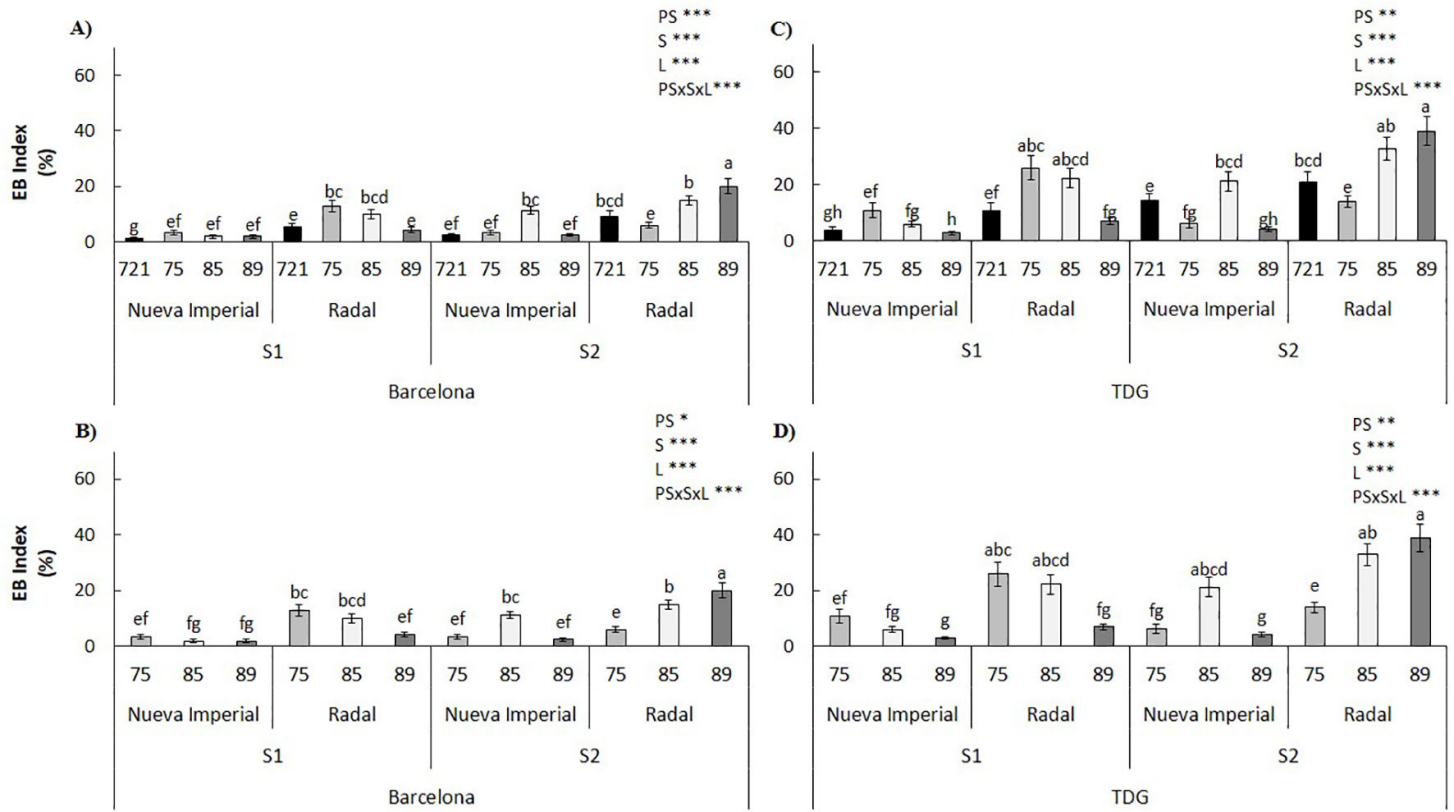
Incidence of enzymatic browning (%) in the shell and kernel of hazelnut cultivars Barcelona and Tonda di Giffoni (TDG), evaluated at different phenological stages according to the BBCH scale (721, 75, 85, and 89) during the 2022/2023 (S1) and 2023/2024 (S2) seasons in Nueva Imperial and Radal, La Araucanía Region, Chile. **(A)** Barcelona-shell; **(B)** Barcelona-kernel **(C)** TDG-shell; **(D)** TDG-kernel. The abbreviations correspond to: PS, phenological stage; S, season; L, location. The bars represent the average of three replicates ± SE. Different letters indicate statistically significant differences between structures and phenological stages for the same season. NS: not significant; * p<0.05; ** p<0.01; *** p<0.001.

In the incidence of EB, significant interactions were observed between PS, S and L in both shell and kernel, in the Barcelona and TDG cultivars (p<0.001) (**Figure 4**).

In Barcelona, during S1, in Nueva Imperial, the incidence of EB in the shell was lower in BBCH 721 compared to later phenological stages. In Radal, during S1, the incidence did not show a clear trend among the phenological stages evaluated. During S2, in Nueva Imperial, the incidence was higher in BBCH 85, by 386, 240, and 366% compared to BBCH 721, 75, and 89. In Radal, during S2, EB increased gradually from BBCH 75, reaching its highest values at 89 (**Figure 4A**). In the Barcelona kernel, during S1, in Nueva Imperial, the incidence showed no significant differences between phenological stages. In Radal, during S1, the incidence of EB gradually decreased until reaching its lowest values in BBCH 89. During S2, in Nueva Imperial, the incidence of EB was higher in BBCH 85 by 240 and 366% compared to BBCH 75 and 89. In Radal, during S2, the incidence of EB increased as maturation progressed (**Figure 4B**).

In TDG, during S1, in Nueva Imperial, the incidence of EB in shell gradually decreased until BBCH 89. In Radal, during S1, the incidence was higher in BBCH 75 and 85, by 136 and 103% compared to BBCH 721, and by 271 and 219% compared to BBCH 89. During S2, in Nueva Imperial, the incidence was higher at BBCH 85, with no clear trend among the stages. In Radal, during S2, the incidence of EB increased progressively from BBCH 75 until ripening (**Figure 4C**). In the TDG kernel, during S1, in Nueva Imperial and Radal, EB incidence decreased progressively with ripening. During S2, in Nueva Imperial, the highest incidence of EB was recorded BBCH 85, at 237 and 392% compared to BBCH 75 and 89. In Radal, during S2, EB incidence showed a progressive increase with maturation (**Figure 4D**).

**Figure 5:**
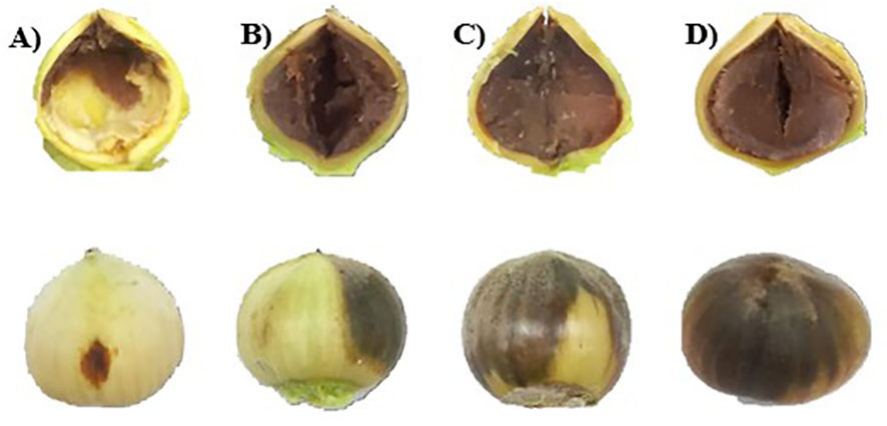
Evolution of enzymatic browning in the kernel and shell throughout four phenological stages of development according to the BBCH scale: **(A)** 721; **(B)** 75; **(C)** 85; and **(D)** 89. The images show progressive changes in the intensity of enzymatic browning during the 2022/2023 (S1) and 2023/2024 (S2) seasons in Radal and Nueva Imperial, La Araucanía Region, Chile.

**Figure 5** provides clear visual evidence of the dynamics of browning in the shell and kernel of hazelnuts throughout the phenological development of the nut. As ripening progresses, from BBCH 721 to the advanced stage BBCH 89, a consistent increase in the intensity of browning is observed, with different manifestations between the two structures. These visual patterns complement and reinforce the quantitative results obtained for the incidence of EB.

### 3.4 Total phenolic content

**Figure 6:**
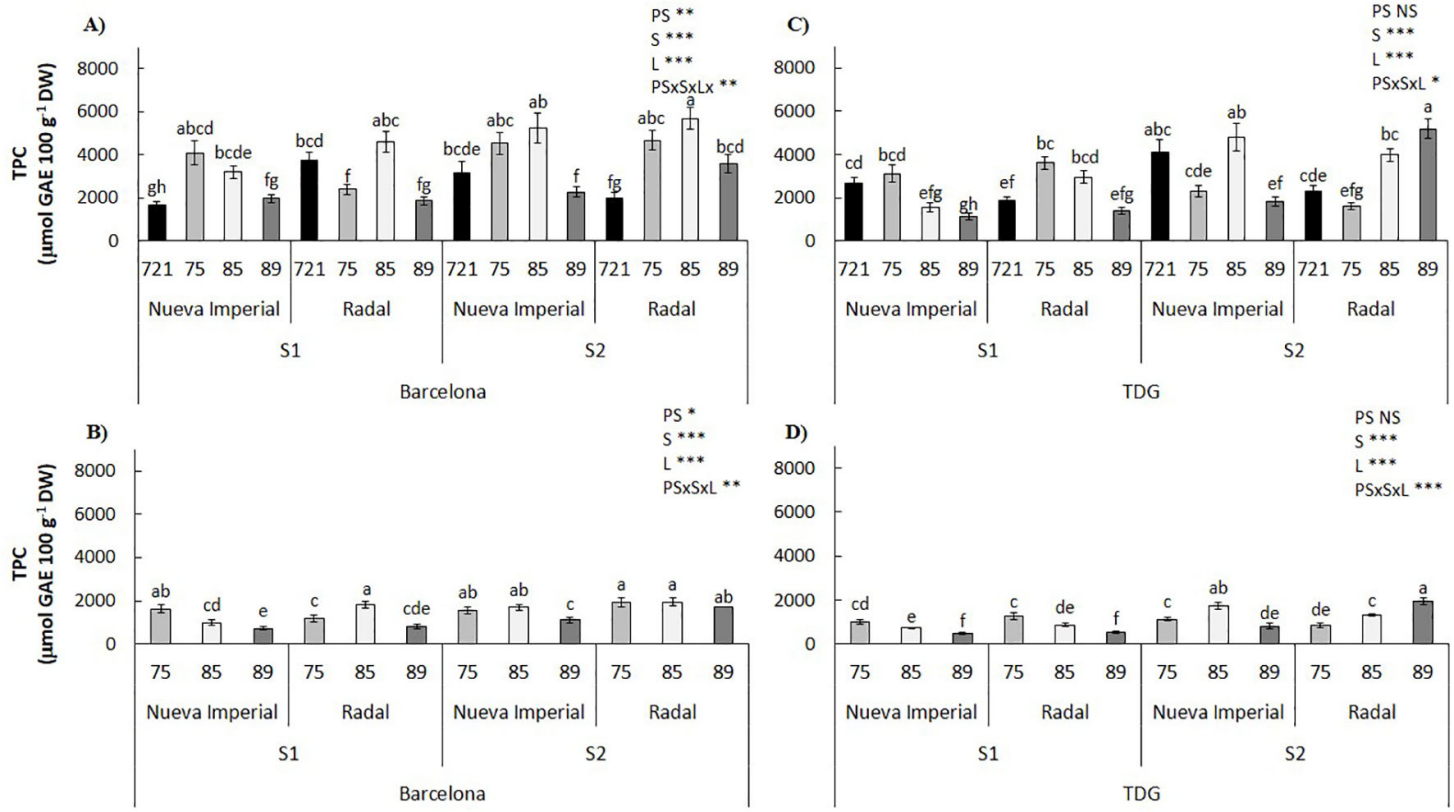
Total phenolic content (μmol GAE 100 g^−1^ of DW) in the shell and kernel of hazelnut cultivars Barcelona **(A)** and Tonda di Giffoni (TDG) **(B)**, evaluated at different phenological stages according to the BBCH scale (721, 75, 85, and 89) during the 2022/2023 (S1) and 2023/2024 (S2) seasons in Nueva Imperial and Radal, La Araucanía Region, Chile. **(A)**) Barcelona-Shell; **(B)** Barcelona-Kernel **(C)** TDG-shell; **(D)** TDG-kernel. The abbreviations correspond to: PS, phenological stage; S, season; L, location. The bars represent the average of three replicates ± SE. Different letters indicate statistically significant differences between structures and phenological stages for the same season. NS: not significant; * p<0.05; ** p<0.01; *** p<0.001.

Total phenolic content (TPC), showed the following interactions in the shell, significant interactions between PS, S, and L in Barcelona (p<0.01) and TDG (p<0.05). In the kernel, significant interactions were detected between PS, S, and L in Barcelona (p<0.01) and TDG (p<0.001) (**Figure 6**).

In Barcelona shell, the TPC showed significant differences in most cases, without showing a clear trend associated with phenological stages, regardless of location and season (**Figure 6A**). In the Barcelona kernel, during S1, in Nueva Imperial, the TPC was higher at BBCH 75 exceeding BBCH 85 and 89 by at 63 and 123%, respectively. In Radal, during S1, the TPC was higher at BBCH 85, exceeding BBCH 75 and 89 by 51 and 120%, respectively. During S2, in Nueva Imperial, the TPC level decreased at BBCH 89 compared to the other stages. In Radal, during S2, there were no statistical differences between phenological stages (**Figure 6B**).

In TDG, during S1, in Nueva Imperial, the TPC in shell was higher in the early stages (BBCH 721 and 75), decreasing in BBCH 85 and reaching the lowest values in BBCH 89. In Radal, during S1, the TPC increased from BBCH 721, with higher values at BBCH 75 and 85, and a reduction at BBCH 89. During S2, in Nueva Imperial, the TPC did not show a clear trend. In Radal, during S2, the TPC showed a progressive increase from BBCH 75 to maturity (**Figure 6C**). In the TDG kernel, during S1, both in Nueva Imperial and Radal, the TPC decreased progressively ripening progressed. During S2, in Nueva Imperial, BBCH 85 showed higher TPC values by 54 and 115% compared to BBCH 75 and 89. In Radal, during S2, TPC increased as ripening progressed (**Figure 6D**).

### 3.5 Enzymatic activity

**Figure 7:**
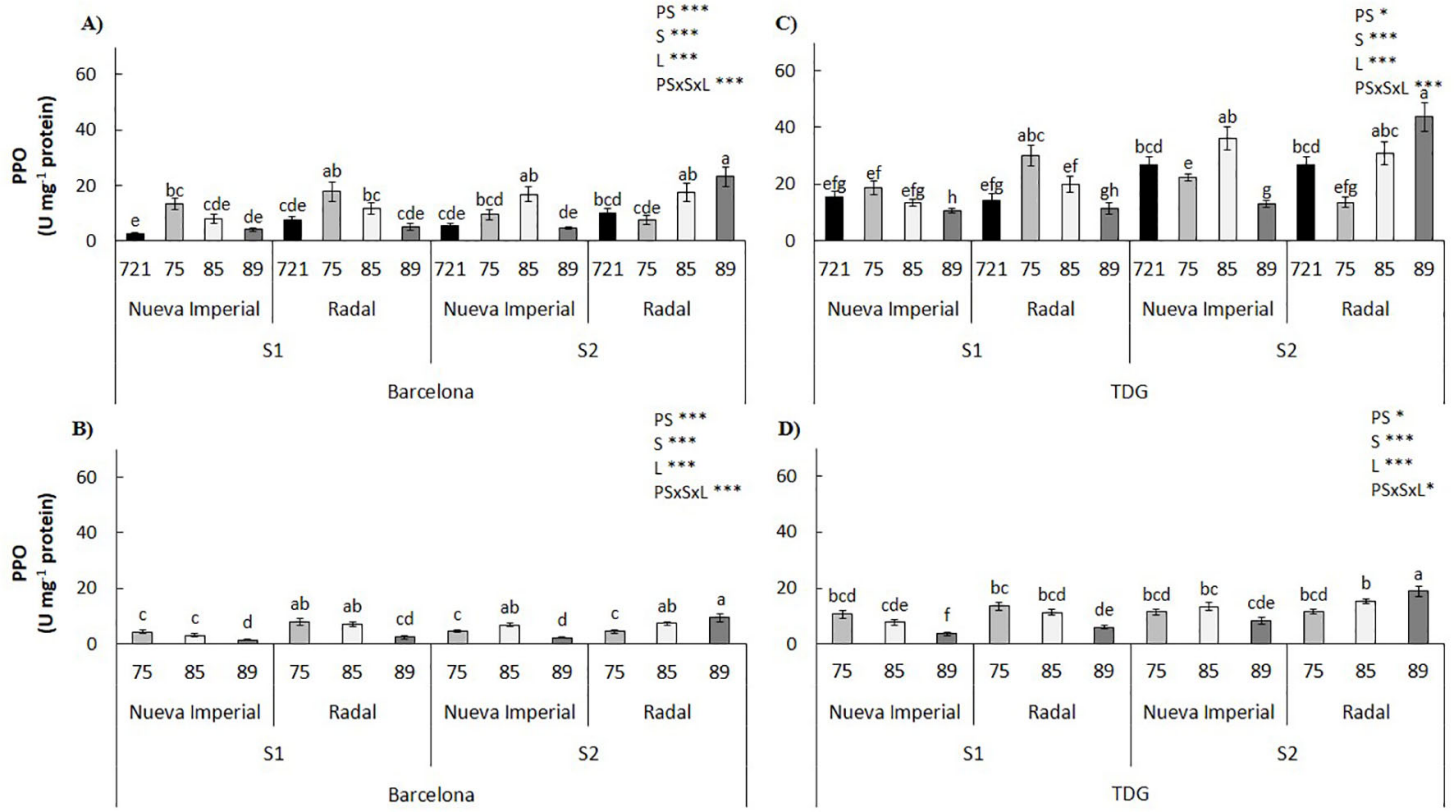
PPO activity (U mg^-1^ protein) in the shell and kernel of hazelnut cultivars Barcelona and Tonda di Giffoni (TDG), evaluated at different phenological stages according to the BBCH scale (721, 75, 85, and 89) during the 2022/2023 (S1) and 2023/2024 (S2) seasons in Nueva Imperial and Radal, La Araucanía Region, Chile. **(A)** Barcelona-shell; **(B)** Barcelona-kernel **(C)** TDG-shell; **(D)** TDG-kernel. The abbreviations correspond to: PS, phenological stage; S, season; L, location. The bars represent the average of three replicates ± SE. Different letters indicate statistically significant differences between structures and phenological stages for the same season. NS: not significant; * p<0.05; ** p<0.01; *** p<0.001.

Polyphenol oxidase (PPO) activity, the following interactions were observed, in the shell, interactions were significant between PS, S, and L in Barcelona and TDG (p<0.001). In the kernel, significant interactions were detected between PS, S, and L in Barcelona (p<0.001) and TDG (p<0.05) (**Figure 7**).

In Barcelona, during S1, in Nueva Imperial, PPO activity in the shell increased by 80% at BBCH 75 compared to BBCH 721, while BBCH 85 and 89 showed no significant differences. In Radal, during S1, PPO activity decreased progressively from BBCH 75 onwards as ripening progressed. During S2, in Nueva Imperial, PPO activity did not show a clear trend among the phenological stages evaluated. In Radal, during S2, PPO activity increased progressively from BBCH 75 to BBCH 89 (**Figure 7A**). In the Barcelona kernel, during S1, both in Nueva Imperial and Radal, PPO activity showed a tendency to decrease from BBCH 75 onwards as ripening progressed. During S2, in Nueva Imperial, PPO activity increased at BBCH 85, being 50% higher than at BBCH 75 and 188% higher than at BBCH 89. In Radal, during S2, PPO activity showed an increasing trend towards harvest (**Figure 7B**).

In TDG, during S1, both in Nueva Imperial and Radal, PPO activity decreased progressively from BBCH 75 until maturity. During S2, in Nueva Imperial, PPO activity did not show a clear trend between phenological stages. In Radal, during S2, values increased progressively from BBCH 75 to maturity (**Figure 7C**). In the TDG kernel, during S1, in both locations, PPO activity showed a decreasing trend as ripening progressed. During S2, in Nueva Imperial, PPO activity did not show statistical differences between phenological stages. In Radal, an increase associated with ripening was observed (**Figure 7D**).

**Figure 8:**
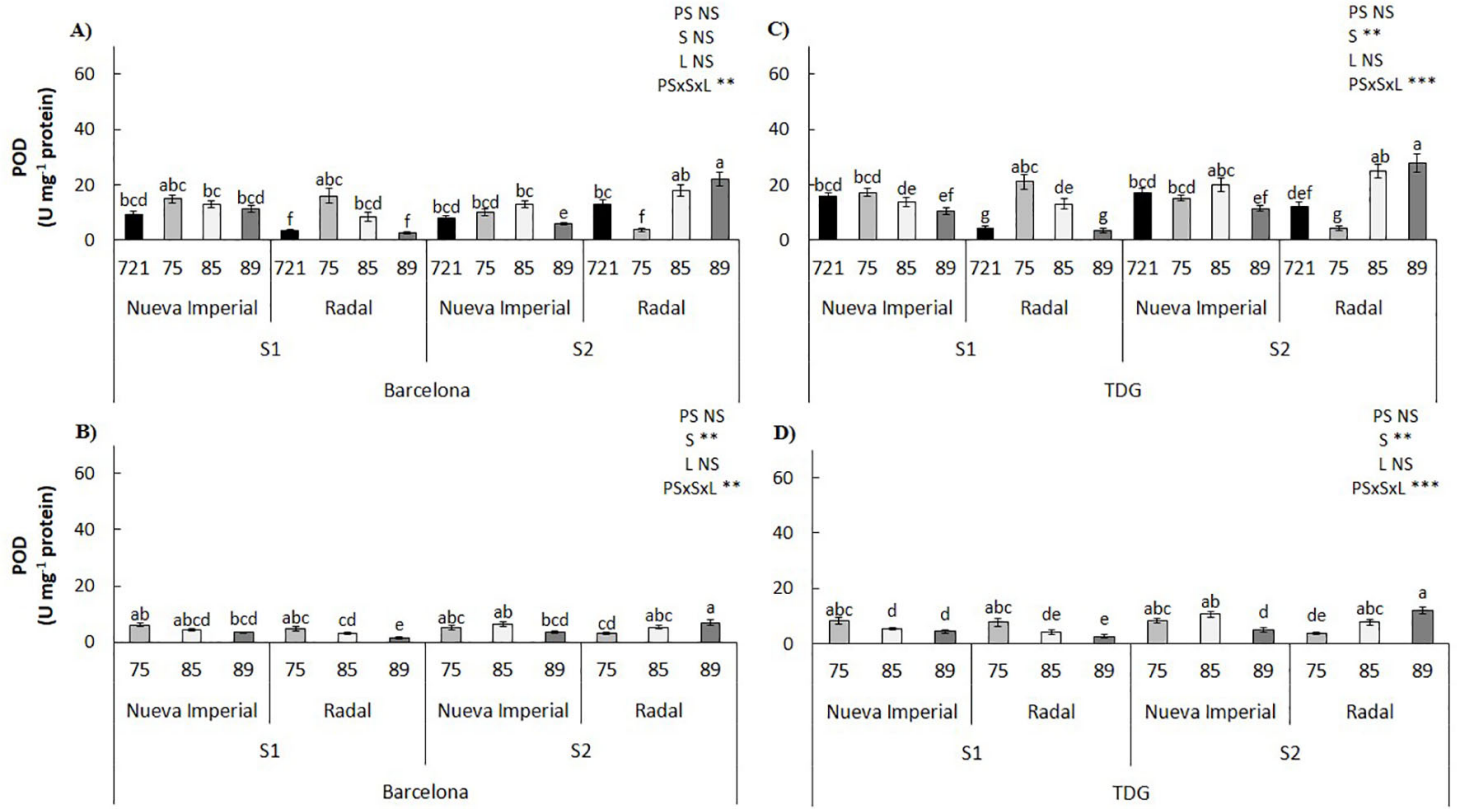
POD activity (U mg^-1^ protein) in the shell and kernel of hazelnut cultivars Barcelona and Tonda di Giffoni (TDG), evaluated at different phenological stages according to the BBCH scale (721, 75, 85, and 89) during the 2022/2023 (S1) and 2023/2024 (S2) seasons in Nueva Imperial and Radal, La Araucanía Region, Chile. **(A)** Barcelona-shell; **(B)** Barcelona-kernel **(C)** TDG-shell; **(D)** TDG-kernel. The abbreviations correspond to: PS, phenological stage; S, season; L, location. The bars represent the average of three replicates ± SE. Different letters indicate statistically significant differences between structures and phenological stages for the same season. NS: not significant; * p<0.05; ** p<0.01; *** p<0.001.

Peroxidase (POD) activity, in the shell and kernel, interactions were significant between PS, S, and L in Barcelona (p<0.01) and TDG (p<0.001) (**Figure 8**).

In Barcelona, during S1, in Nueva Imperial, POD activity in the shell showed a decreasing trend from BBCH 75 to maturity. In Radal, during S1, the highest POD value was observed at BBCH 75, decreasing progressively as maturity progressed. During S2, in Nueva Imperial, POD activity showed an increasing trend from BBCH 721 to BBCH 85. In Radal, during S2, POD activity showed a progressive increase from BBCH 75 until ripening (**Figure 8A**). In the Barcelona kernel, during S1, in Nueva Imperial, POD activity showed no statistical differences between the other phenological stages. In Radal, during S1, POD activity decreased as ripening progressed. During S2, in Nueva Imperial, POD activity showed no definite trend. In Radal, during the same season, it increased progressively as ripening progressed (**Figure 8B**).

In TDG, during S1, in Nueva Imperial, POD activity in the shell showed a decreasing trend from BBCH 75 to maturity. In Radal, during S1, the highest POD activity was observed at BBCH 75, with increases of 410, 60, and 529% compared to BBCH 721, 85, and 89, respectively. During S2, in Nueva Imperial, POD activity did not show a clear trend among the phenological stages evaluated. In Radal, during S2, POD activity showed an increasing trend from BBCH 75 to maturity (**Figure 8C**). In the kernel of TDG, during S1, in Nueva Imperial, POD activity was higher at BBCH 75 than at BBCH 85 and 89, with increases of 48 and 84%, respectively. In Radal, during S1, POD activity showed a decreasing trend with advancing maturity. During S2, in Nueva Imperial, POD activity was higher at BBCH 75 and 85, with increases of 61 and 109% compared to BBCH 89. In Radal, during S2, POD activity showed an increasing trend associated with the progress of maturation (**Figure 8D**).

### 3.6 Multivariate analysis

**Figure 9:**
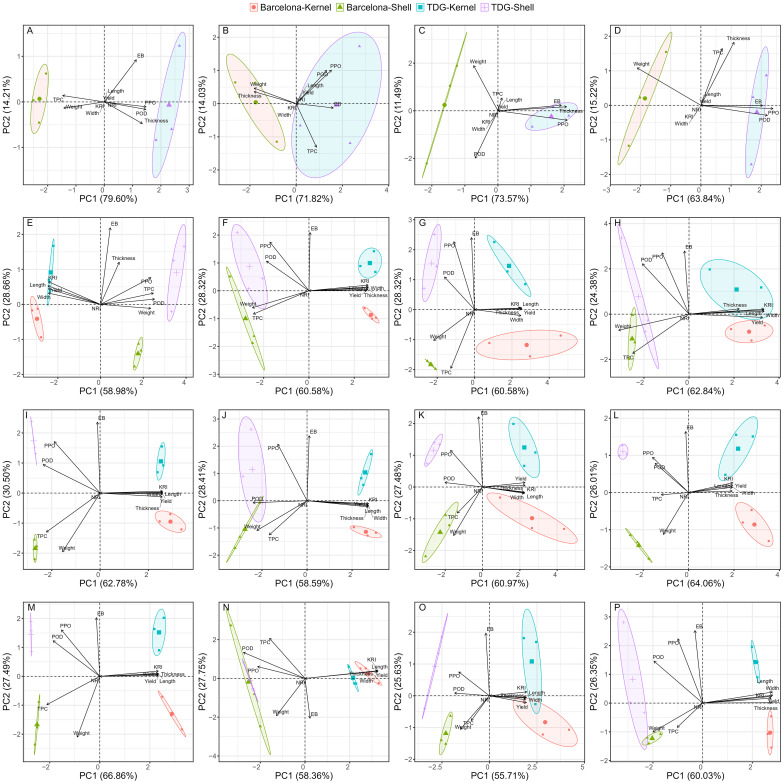
Biplot of the principal component analysis (PCA) of the physicochemical variables in the shell and kernel of hazelnuts (*Corylus avellana* L.), cultivars Barcelona and Tonda di Giffoni (TDG), evaluated at different phenological stages according to the BBCH scale (721, 75, 85, and 89) during the 2022/2023 (S1) and 2023/2024 (S2) seasons in Nueva Imperial (NI) and Radal (R), La Araucanía Region, Chile. Each panel corresponds to a specific phenological stage, season, and location: **(A)** 721-S1-R; **(B)** 721-S1-NI; **(C)** 721-S2-R; **(D)** 721-S2-NI; **(E)** 75-S1-R; **(F)** 75-S1-NI; **(G)** 75-S2-R; **(H)** 75-S2-NI; **(I)** 85-S1-R; **(J)** 85-S1-NI; **(K)** 85-S2-R; **(L)** 85-S2-NI; **(M)** 89-S1-R; **(N)** 89-S1-NI; **(O)** 89-S2-R; **(P)** 89-S2-NI. The vectors indicate the contribution of physicochemical variables, including TPC, total phenolic content; PPO, polyphenol oxidase; POD, peroxidase; EB, enzymatic browning; weight; length; thickness; width; KRI, kernel roundness index; NRI, nut roundness index; yield.

The principal component analysis (PCA) presented in **Figure 9** allowed us to integrate and visualize the relationship between the physicochemical variables evaluated in the shell and kernel of the Barcelona and TDG cultivars, considering phenological stages, seasons, and locations.

In phenological stage 721 (panels A–D), the first principal component (PC1) explained between 64–80% of the variability and the second component (PC2) between 12–15%, showing that the separation of samples was mainly determined by differences between structural and oxidative variables. In Radal S1 (Panel A), the Barcelona shell was located in the quadrant associated with morphological variables and TPC, while in Nueva Imperial S1 (Panel B), the Barcelona shell was mainly related to physical characteristics (weight, width, length, KRI), indicating lower stress. In S2 in Radal (Panel C), the TDG shell showed a greater association with vectors linked to EB and oxidative enzymes. In Nueva Imperial S2 (Panel D), Barcelona was grouped with structural variables, indicating a more stable physiological condition, while TDG was associated with enzymatic activities.

In stage 75 (panels E–H), PC1 explained 59–63% and PC2 24–29%, reflecting the joint contribution of oxidative and structural traits. In Radal S1 (Panel E), the shell and kernel of Barcelona shifted towards TPC and structural variables, although without an extreme association, while the shell of TDG projected strongly towards PPO and POD. In Nueva Imperial S1 (Panel F), both cultivars remained close to the structural variables, although TDG shell showed an approach to PPO and POD. In Radal S2 (Panel G), TDG shell and kernel showed the highest oxidative load (PPO, POD, and EB) in stage 75, while Barcelona showed a moderate shift. In Nueva Imperial S2 (Panel H), both Barcelona structures were mainly associated with TPC and some structural variables, indicating a less stressful scenario, while the TDG shell was associated with PPO and POD.

In stage 85 (panels I–L), PC1 explained 59–64% and PC2 29–31%, showing a balance between structural and biochemical variables, although with greater oxidative expression in adverse environments. In Radal S1 (Panel I), Barcelona began to relate more closely to structural variables and TPC, while TDG aligned directly with PPO and POD, confirming its sensitivity. In Nueva Imperial S1 (Panel J), both cultivars clustered around structural variables, especially weight, length, and NRI, confirming more favorable conditions. In Radal S2 (Panel K), TDG shell and kernel showed the most extreme shift towards the oxidative axis, being one of the most decisive PCA to highlight their vulnerability, while Barcelona moved towards structural variables and TPC. In Nueva Imperial S2 (Panel L), both cultivars maintained a stable structural pattern.

In phenological stage 89 (panels M–P), PC1 explained between 56–67% and PC2 between 26–28%, with joint contributions from physical and biochemical variables. In Radal S1 (Panel M), Barcelona shell projected towards TPC, while the kernel projected towards weight and yield, showing moderate stress typical of ripening. The TDG shell was even closer to PPO, POD, and EB, reflecting greater vulnerability, while the kernel was associated with yield, length, and thickness (M). In Nueva Imperial S1 (Panel N), the shells of both cultivars were associated with TPC and oxidative enzymes, while the kernel was related to nut dimensions, thickness, and yield. In Radal S2 (Panel O), TDG shell and kernel projected strongly directly towards EB, PPO, and POD, showing their highest oxidative expression in the entire PCA series, while Barcelona showed an intermediate shift. In Nueva Imperial S2 (Panel P), Barcelona kernel and shell retained their structural profile, far from the oxidative vectors, while TDG shell maintained its slight association with PPO and POD. This state confirmed that TDG in Radal S2 is the scenario of greatest susceptibility to browning, characterized by a high accumulation of phenolic substrates and high enzymatic activity under unfavorable agroclimatic conditions.

In general, PCA showed that enzymatic browning is a multifactorial process determined by the interaction between cultivar genetics, nut physiology, and conditions associated with the season and location. Radal was consistently associated with oxidative variables, especially in S2, reflecting a more stressful environment, while Nueva Imperial was grouped with physical-morphological traits, consistent with more favorable nut development. Barcelona maintained a stable position and was mainly associated with structural variables, confirming its lower susceptibility to browning. In contrast, TDG was consistently grouped with stress variables, standing out as the most vulnerable cultivar, especially under adverse conditions such as those observed in Radal S2. These differences became more evident in BBCH 85 and 89, stages naturally more prone to browning due to the greater accumulation of phenolic substrates and increased enzymatic activity.

## 4 Discussion

The agroclimatic variations between Nueva Imperial and Radal during S1 and S2 reveal significant microenvironmental contrasts. In S1, Nueva Imperial experienced winter precipitation and high relative humidity, with a gradual transition to drier summer conditions, which would have reduced abiotic stress during BBCH 75-89, attenuating the oxidative processes associated with EB by limiting cell dehydration and oxygen availability in tissues (Zhou et al. 2015). In contrast, Radal had higher precipitation, concentrated mainly in winter, with additional precipitation in autumn and spring, along with slightly higher maximum temperatures and radiation levels. Although these conditions could have created a more demanding environment, the greater water availability would have partially mitigated water stress and limited EB, considering that its increase is mainly associated with water deficit combined with high radiation (Wang et al., 2024). In S2, Nueva Imperial presented a relatively balanced climate pattern, with winter precipitation, high relative humidity in the cold months, and a seasonal increase in radiation and temperature in summer. Despite maximum temperatures above 26 °C, the gradual transition to drier conditions would have favored the physiological stability of the nut and attenuated the oxidative processes associated with EB, according to Zhou et al. (2015). In contrast, Radal presented the most restrictive conditions in the study, with precipitation concentrated in winter, water deficit in summer, lower humidity, and higher values of solar radiation and temperature. This combination would have intensified the water deficit, generating greater abiotic stress during the final stages of nut development (BBCH 85 to 89), favoring cell destabilization and enzymatic oxidation associated with EB, according to Xu et al. (2022) and Wang et al. (2024).

The industrial parameters of the nut, shell, and kernel show the impact of EB on the structural integrity and physical quality of the hazelnuts. In Barcelona during S1, Nueva Imperial nuts showed high physical and industrial quality, associated with a balanced morphology. The shell showed a more compact and protective structure, suggesting greater structural firmness, while the kernel showed adequate filling during development, with no significant changes in its dimensions, KRI, or yield. Taken together, these characteristics indicate a favorable morphology that could contribute to a lower incidence of EB by preserving cell integrity during ripening (Gavilán-CuiCui et al., 2025). In Radal, the nuts and kernels from Barcelona showed progressive and morphologically stable development, with adequate structural protection of the shell and consistent physical variables. This pattern indicates homogeneous growth and is consistent with studies that highlight the interaction between environment and genotype in the industrial quality of nuts (Gavilán-CuiCui et al., 2024). During S2 in Nueva Imperial, Barcelona nuts showed restricted initial development (BBCH 721), with partial recovery towards maturity (BBCH 89), along with increased shell thickness, suggesting an adaptive response associated with lignification as described by Toillon et al. (2023). In the kernel, limited filling in the early stages was compensated for in the final stages without affecting yield, demonstrating physiological plasticity in response to stress (McCauley et al., 2024; Moine et al., 2024; Gavilán-CuiCui et al., 2024). In Radal, nut development was more limited, with insufficiently consolidated shells and kernels and incomplete recovery, associated with water deficit and high temperatures, which could compromise structural integrity and increase susceptibility to EB (Freitas et al., 2021; Calvo et al., 2023). In TDG during S1 in Nueva Imperial, the nut showed progressive development and consistent evolution of the shell, with stability in key morphological variables. At the kernel level, weight increased towards maturity while other characteristics remained stable, reflecting homogeneous nut growth, which could contribute to limiting EB in the early stages (Romero et al., 2001, Romero et al., 2003). In Radal, the nuts also showed progressive growth, with stability in NRI and shell thickness. At the kernel level, the increase in weight and yield towards maturity, together with the stability of its dimensions and KRI, indicates homogeneous development. Although TDG did not show severe limitations under these conditions, previous studies point to its greater sensitivity to water and heat stress scenarios (McCauley et al., 2024; Durán et al., 2025), which could influence susceptibility to EB (Ortiz et al., 2019). During S2 in Nueva Imperial, TDG showed limited initial development of the nut and kernel, although toward maturity there was a partial recovery of the nut and stability in the NRI and shell thickness, the increase in kernel weight, yield, and KRI did not compensate for the smaller size compared to S1, indicating that S2 conditions restricted full development and caused late and incomplete filling, which increased susceptibility to EB (Durán et al., 2025). In Radal, TDG showed delayed initial development of the nut and shell, and small, underdeveloped kernels, resulting in less robust nuts that were more susceptible to EB under the climatic conditions of the season (Calvo et al., 2023; Yildiz and Karaca, 2024; Durán et al., 2025).

The incidence of EB was influenced by interactions between different factors, reflected in both the shell and the kernel. In Barcelona, during S1, in Nueva Imperial, the low incidence of EB in the shell at BBCH 721 was associated with moderate environmental conditions and the structure of the cultivar, which favored more homogeneous ripening and limited the activation of oxidative processes. In this context, the kernel maintained a stable response between phenological stages, attributable to adequate synchrony between its growth and the protection conferred by the shell, in agreement with Gavilán-CuiCui et al. (2024). In Radal, the shell did not show a clear EB trend between phenological stages, while in the kernel, the progressive decrease in EB towards maturity could be related to greater structural organization of the tissue and less exposure of oxidizable substrates (Gavilán-CuiCui et al., 2025). During S2, in Nueva Imperial, the highest incidence of EB at BBCH 85 reflected a phase of high sensitivity in both structures, coinciding with intense metabolism and accelerated growth prior to complete structural consolidation (Gavilán-CuiCui et al., 2025). In Radal, the increase in EB towards maturity in the shell and kernel suggests that water deficit and high radiation compromise the tissue integrity of the nut, favoring microdamage and contact between phenolic compounds and oxidative enzymes, even in firm-shell cultivars, as previously reported by Cristofori et al. (2012) and McCormick et al. (2021). In TDG, during S1 in Nueva Imperial, the incidence of EB decreased progressively with maturity in both structures, a behavior associated with the structural characteristics of the cultivar, particularly a thinner shell that favors differential dehydration, tissue stress, and less protection of the internal tissue, increasing susceptibility to breakage and EB, in accordance with what has been described for thin-shell cultivars (Pacchiarelli et al., 2024). In Radal, the higher incidence of EB in the shell at BBCH 75 and 85 indicated high sensitivity during phases of intense metabolic activity, where radiation and thermal stress enhance structural alterations and early oxidative processes (Gavilán-CuiCui et al., 2025), while in the kernel, the progressive reduction of EB with maturation was associated with its more porous structure and limited shell protection (Pacchiarelli et al., 2024). During S2, in Nueva Imperial, the increase in EB at BBCH 85 in both tissues revealed critical stages of vulnerability linked to elevated metabolism and a structure that had not yet stabilized (Gavilán-CuiCui et al., 2025). In Radal, the increase in EB towards maturity in the shell and kernel confirms that hot and dry conditions accelerate tissue deterioration, reinforcing the greater susceptibility of TDG under extreme environments, consistent with observations by Sidhu et al. (2023).

The TPC concentration, an indicator of phenolic content, was influenced by interactions between different factors, reflected in both the shell and the kernel. In Barcelona during S1, Nueva Imperial, the shell reached higher TPC levels in intermediate stages, indicating that under moderate conditions, phenolic accumulation is concentrated in phases of high metabolic activity, without a strictly phenological pattern (Serra et al., 2021; Di Michele et al., 2021), while in the kernel, TPC decreased towards maturity, showing greater phenolic stability. In Radal, both structures showed increases in TPC with maxima at BBCH 721 and 85, associated with microclimatic fluctuations and accumulated environmental stress (Kumar et al., 2023; Wang et al., 2025). During S2, in Nueva Imperial, the shell and kernel showed high TPC levels in the early and intermediate stages, with a reduction towards maturity, suggesting greater phenolic stability under less stressful conditions (Najjari and Bodaghi, 2024). In Radal, the shell showed transient phenolic activation in intermediate stages under greater stress, favoring oxidative processes associated with EB (Persic et al., 2017; Wang et al., 2023; Yang et al., 2024), while no relevant variations were observed in the kernel between stages. In TDG during S1 in Nueva Imperial, the shell showed higher TPC levels at BBCH 721 and 75, suggesting lower oxidation in early stages, while in the kernel, TPC decreased towards maturity, indicating that phenolic conservation is favored in early stages of development, as reported by Najjari and Bodaghi (2024). In Radal, the progressive accumulation of TPC in the shell at BBCH 75 and 85 reflects a compensatory response to early environmental stress, associated with the activation of the phenylpropanoid pathway in more sensitive cultivars (Gavilán-CuiCui et al., 2024), while in the kernel, the reduction in TPC towards maturity suggests phenolic degradation and increased oxidative activity, consistent with susceptibility of TDG to stress (Gavilán-CuiCui et al., 2024; Persic et al., 2017). During S2 in Nueva Imperial, the shell did not show a defined phenological pattern, while in the kernel, higher TPC levels at BBCH 85 suggest greater phenolic stability. In Radal, the shell showed a sustained increase in TPC until maturity associated with stress and oxidative damage, as has been described in macadamia and walnuts under high environmental demand (Srichamnong and Srzednicki, 2015; Guo et al., 2020), while in the kernel this increase was linked to a higher incidence of EB and severe oxidative damage, as described by Zhao et al. (202) and Serra et al. (2021).

PPO and POD activity showed interactions between factors in the shell and kernel, reflecting enzyme dynamics. In Barcelona, during S1 in Nueva Imperial, PPO in the shell increased at BBCH 75 compared to BBCH 721, with no subsequent changes, while in the kernel it decreased with maturation, reflecting the influence of the microenvironment in early stages (Sultan et al., 2025). POD in the shell decreased from BBCH 75 until maturity and remained stable in the kernel. In Radal, PPO activity decreased progressively from BBCH 75 to maturity in the shell and kernel, indicating greater control of oxidative processes in the final stages and a lower incidence of EB as reported by Gheysarbigi et al. (2020), with the influence of the microenvironment in the early stages of development (Sultan et al. (2025). POD activity also decreased from BBCH 75 to maturity in both structures, showing initial antioxidant activation associated with phases of high metabolic activity and regulated by phenology and climatic conditions of the season and location (Gavilán-CuiCui et al., 2025 and Lu et al., 2025). During S2, in Nueva Imperial, PPO activity in the shell did not show a clear trend between phenological stages, while in the kernel it increased at BBCH 85, reflecting oxidative activation associated with accumulated environmental stress (Wang et al., 2024). POD activity in the shell increased from BBCH 721 to BBCH 85, consistent with its stimulation under stress (Wang et al., 2024), while in the kernel it did not show a definite trend, suggesting regulation dependent on the interaction between phenology and local climatic conditions (Lu et al., 2025). In Radal, both enzymes increased progressively in the shell and kernel from BBCH 75 to maturity, reflecting an intensification of oxidative processes induced by demanding environmental conditions and increased susceptibility to EB (Wang et al., 2024; Sultan et al., 2025; Eljebbawi et al., 2022). In TDG, during S1 in Nueva Imperial, PPO in the shell and kernel decreased progressively from BBCH 75 towards maturity, suggesting a reduction in the availability of oxidizable substrates and an attenuation of processes associated with EB due to structural consolidation as described by Gavilán-CuiCui et al. (2025). POD in the shell also decreased with maturation, while in the kernel it was higher at BBCH 75, evidencing a regulation dependent on development and stress intensity (Ma et al., 2021). In Radal, a similar pattern was observed for PPO in both structures, and early activation of POD in the shell, with a decreasing trend in the kernel, associated with initial antioxidant responses to environmental stress (Ma et al., 2021). During S2 in Nueva Imperial, PPO did not show a definite trend in the shell and remained stable in the kernel, suggesting efficient enzymatic control that is less dependent on phenology under moderate microenvironmental conditions (Zhao et al., 2021; Zhang, 2023). POD showed greater variability between structures, evidencing compensatory antioxidant mechanisms (Sultan et al., 2025). In Radal, both PPO and POD increased progressively in the shell and kernel toward maturity, reflecting intensified environmental stress and greater susceptibility to EB, as reported by Wang et al. (2024) and Gavilán-CuiCui et al. (2025).

Overall, the results indicate that EB in hazelnuts is a multifactorial phenomenon rather than a response to a single dominant factor. Environmental variations between locations and seasons influenced the balance between nut structural development and the regulation of oxidative and antioxidant mechanisms, especially at critical phenological stages. In this context, Barcelona showed greater physical-biochemical stability and adaptability to variable environmental conditions, which was reflected in lower susceptibility to EB, while TDG showed greater sensitivity to water deficit and high radiation scenarios. Thus, the incidence of EB was mainly linked to imbalances in the synchrony between nut structure, phenolic content, and enzymatic activity, rather than to a direct and isolated effect of climatic stress.

## 5 Conclusion

EB in hazelnuts is a multifactorial process resulting from the interaction between physiology and seasonal and local conditions, regulated by the synchrony between the physical integrity of the nut, the content of phenolic compounds, and the activity of PPO and POD enzymes. The comparative analysis between cultivars showed contrasting responses, with Barcelona exhibiting greater physical-biochemical stability and lower incidence of EB in the shell and kernel, especially in Nueva Imperial and in both seasons, while TDG showed greater susceptibility, especially in Radal during S2 and in advanced phenological stages (BBCH 85–89), associated with higher levels of environmental stress and enzyme activation. Taken together, these results provide key information for varietal selection and agronomic management of hazelnuts in Chile. As a projection, there is a need to further identify specific phenolic compounds associated with browning tolerance and to develop management strategies aimed at mitigating environmental stress in restrictive locations, contributing to more resilient production systems in the face of climate variability.

## Data Availability

The raw data supporting the conclusions of this article will be made available by the authors, without undue reservation.
